# Lupenone preserves T cell activity by recovery of CD40L expression and protection from cytotoxicity due to methamphetamine exposure

**DOI:** 10.1371/journal.pone.0314054

**Published:** 2025-03-20

**Authors:** Hyun-Su Lee, Eun-Nam Kim, Gil-Saeng Jeong

**Affiliations:** 1 Department of Physiology, Daegu Catholic University School of Medicine, Daegu, Republic of Korea; 2 College of Pharmacy, Chungnam National University, Daejeon, Republic of Korea; Rutgers: Rutgers The State University of New Jersey, UNITED STATES OF AMERICA

## Abstract

Methamphetamine (METH) is one of the most highly compulsive drugs in the world and has become a major public health problem over the last two decades. Exposure to METH has been investigated to cause neuronal toxicity but little is known about the effect of METH on the activity and toxicity of T lymphocytes. Lupenone has been reported to possess anti-diabetic, anti-inflammatory and anti-apoptotic effects but little is known about whether lupenone has a protective effect on T cell activation in METH-exposed cells. We evaluated the cytotoxicity and cytoprotective effects of lupenone in METH-stimulated Jurkat T cells. Results from the inhibitor assay using CD40L blocking antibodies revealed that this was due to enhanced CD40L expression on the T cells by pre-treatment with lupenone. Pre-treatment with lupenone significantly reduces METH-induced toxicity by restoring the expression of anti-apoptotic proteins in activated T cells. The protective effects of lupenone on activated T cells exposed to METH were associated with the prevention of MAPK and PI3K/Akt/mTOR pathways. These data suggest lupenone protected T cell activity by elevating CD40L expression and cell viability in cells exposed to methamphetamine. Our data showed that lupenone treatment recovered the expression of IL-2 and CD69 in METH-exposed cells.

## Introduction

Methamphetamine (METH) has been one of the most abused drugs worldwide in the last two decades. Studies have shown that it is a potent central nervous system (CNS) stimulant [1]. Since METH causes the release of biogenic amines from nerve terminals, it is highly addictive and dangerous to public health [2]. The risk of METH addiction has been demonstrated in neuronal degenerative disorders including Alzheimer’s disease (AD) and Parkinson’s disease (PD) and neuronal cytotoxicity or apoptosis from METH exposure causes AD and PD [3,4]. Several reports have revealed that METH affects both the innate and adaptive immune systems, resulting in insufficient phagocytosis by dendritic cells, enhanced HIV infectivity, and changes in gene expression in immune cells [5–7]. T lymphocytes are considered central players in increasing immune responses by bridging innate and adaptive immunity. Recent studies investigating how METH affected T cell activity and function reported that METH exposure regulated the cell cycle entry of T lymphocytes and its progression [8], promoted infectivity by retroviruses through immuno-suppression [9], and inhibited the numbers of CD4 + T cells in drug-addicted animal models. Thus, METH has several effects on T cell activity, but little is known about the deleterious effect of METH exposure on T cell activation and cytotoxicity.

The activation of naïve T lymphocytes is initiated in the typical interaction with antigen-presenting cells (APCs) including dendritic cells, macrophages, and B lymphocytes, in an interface called the immunological synapse [10]. It is tightly regulated by several molecules expressed on cell surfaces to prevent abnormal responses. CD40 ligand (CD40L), also called CD154, is a co-stimulatory surface molecule expressed on naïve T cells but gradually induced on activated T cells that plays a critical role in regulating T cell activation by triggering specific pathway [11]. In early phase of T cell activation, CD40L is involved in providing a stimulatory signal for T cell priming but progressively expressed CD40L on activated T cells leads to promote B cell differentiation and class switching in late phase of T cell activation [12]. However, few studies have reported whether the expression of CD40L was inhibited in METH exposed T cells and the effect of METH on the formation of immunological synapses with APCs, even though CD40L is a highly pivotal molecule for T cell-mediated immune responses.

Lupenone, one of the triterpenes of lupane, is a natural bioactive molecule found in several plants, such as *Sorbus lanta Schauer, Anadenanthera colubrina Brenan, Corbus commixta Hedl* and *Sorbus commixta* [13, 14]. Among them, it has been explored that lupenone and lupeol are major constituents of *S. commicta* which is traditionally used to treat liver diseases, asthma and pulmonary tuberculosis in Korea [15, 16]. On the other hand, lupenone has been shown to possess multiple bioactivities including anti-diabetic, anti-inflammatory, and anti-cancer activities [17–19]. Recently, our group reported the anti-apoptotic effect of lupenone on SH-SY5y cells, showing that lupenone suppressed neuronal cytotoxicity induced by METH exposure via the PI3K/Akt/mTOR pathway [20]. Treatment with lupenone has been shown to increase the expression of anti-apoptotic proteins and signaling molecules involved in anti-apoptotic effects on neuroblastoma SH-SY5y cells in the presence of METH. However, a protective effect of lupenone on the activity and viability of T lymphocytes exposed to METH has not been shown. In the present study, we explored the effect of lupenone to prevent T cell activation via the upregulation of CD40L expression. Moreover, we investigated whether pre-treatment with lupenone inhibited the cytotoxicity induced by METH exposure through the upregulation of anti-apoptotic protein expression.

## Materials and methods

### Cells

Jurkat T cells and Raji B cells were obtained from the Korean Cell Line Bank (Seoul, Republic of Korea). The cells were cultured in RPMI medium (Welgene, Gyeongsan, Republic of Korea) supplemented with 10% fetal bovine serum (FBS), penicillin G (100 units/mL), streptomycin (100 μg/mL), and L-glutamine (2 mM). The cells were grown at 37°C in a humidified incubator containing 5% CO_2_ and 95% air.

### Reagents and antibodies

MTT (1-(4,5-dimethylthiazol-2-yl)-3,5-diphenylformazan) powder, METH powder, phorbol 12-myristate 13-acetate (PMA), and A23187 were obtained from Sigma Chemical Co. (St. Louis, MO, USA). Annexin-V dye for the IncuCyte^®^ cell imaging system was purchased from Essen Bio (Ann Arbor, MI, USA). ECL Western blotting detection reagents, an NE-PER Nuclear and Cytoplasmic Extraction Reagent Kit and CMRA and CMFDA cell trackers were obtained from Thermo Fisher Scientific (Waltham, MA, USA). The annex-in-V/propidium iodide (PI) apoptosis assay kit was purchased from BD Biosciences (San Diego, CA, USA). The human IL-2 DuoSet^®^ ELISA kit was obtained from R&D Systems (Minneapolis, MN, USA). Staphylococcal enterotoxin E (SEE) was purchased from Toxin Technology (Sarasota, FL, USA). Anti-CD40L neutralizing antibodies were purchased from InvivoGen (San Diego, CA, USA). Anti-Bcl2, anti-CD40L, and anti-phosphorylated mTOR (S2448) antibodies were purchased from Santa Cruz Biotechnology (Dallas, TX, USA). Anti-caspase 3, anti-caspase 9, anti-caspase 8, anti-caspase 7, anti-p65, anti-β-actin, anti-phosphorylated IκBα (S32), anti-IκBα, anti-phosphorylated PI3K (Y458/Y199), anti-PARP, anti-phosphorylated Akt (S473), anti-PI3K, anti-mTOR, anti-Akt, anti-phosphorylated ERK (T202/Y204), anti-phosphorylated p38 (T180/Y182), anti-ERK, anti-phosphorylated JNK (T183/Y185), anti-p38, and anti-JNK antibodies were purchased from Cell Signaling Technology (Danvers, MA, USA). Anti-CD69 and anti-CD40L antibodies conjugated with allophycocyanin (APC) were obtained from eBiosciences (San Diego, CA, USA).

### Isolation of lupenone from *S. commixta*

Lupenone was isolated from the dried stem barks of *S. commixta* as previously reported [14]. A voucher specimen (accession number: CPE-29) was deposited at natural products laboratory, college of pharmacy, Chungnam National University (Daejeon, Korea). Briefly, the dried stems of *S. commixta* (0.5 kg) were extracted in MeOH (1 L) by reflux at 85°C for 2 h and filtered. The MeOH extract (60.5 g) was suspended in H_2_O (500 mL) and then partitioned with EtOAc (0.5 L), n-hexane (0.5 L), and n-butanol (0.5 L). Among them, the EtOAc fraction (10.7 g) was open-column chromatographed on silica gel (6.5 ×  60 cm; 70-230 mesh) using a gradient of *n*-hexane −  CH_2_Cl_2_ (1:3 v/v) to obtain Fr. 1 – 9. Of these, Fr. 5 (2.3 g) was eluted with *n*-hexane −  acetone (5:1 v/v) in Sephadex LH-20 column chromatography and purified by silica gel column elution with n-hexane-MeOH (4:1 v/v) to yield Fr. 5-1. Fraction 5-1 was analyzed by ^1^H and ^13^C-NMR (JEOL JNM-ECA 500) spectroscopy, and the spectral data were identified as lupenone (12 mg) by comparison to the reported literature [14].

Lupenone: ^1^H-NMR (500 MHz, CDCl_3_) δ: 4.70 (1H, m, H-29α), 4.55 (1H, m, H-29β), 2.51 (1H, m), 1.88 (2H, m), 1.65 (3H, s), 1.03 (3H, s), 0.99 (3H, s), 0.94 (3H, s), 0.77 (3H, s). ^13^C-NMR (500 MHz, CDCl_3_) δ: 54.4 (C-5), 49.5 (C-9), 49.2 (C-18), 48.0 (C-19), 47.1 (C-4), 43.1 (C-14), 43.0 (C-17), 40.7 (C-8), 40.0 (C-22), 39.7 (C-1), 38.3 (C-13), 37.0 (C-10), 34.2 (C-2), 35.7 (C-16), 33.8 (C-7), 29.9 (C-21), 27.3 (C-15), 27.0 (C-23), 25.1 (C-12), 21.5 (C-11), 21.3 (C-24), 19.7 (C-6), 19.4 (C-30), 18.4 (C-28), 16.2 (C-25), 16.0 (C-26), 14.8 (C-27).

### MTT assay

Jurkat T cells (1 ×  10^4^/96-well plate) were seeded and treated with the indicated concentrations of lupenone (0 to 40 μM) and/or 2 mM of METH for 24 h. In some cases, Jurkat T cells were treated with anti-CD3/CD28 antibodies for 24 h. The supernatants were re-moved and 500 μg/ml of MTT was added to the cells for 1 h. The supernatants were dis-carded and 150 μL of DMSO was added to dissolve the formazan crystals. The absorbance was read at 540 nm and cell viability was calculated relative to the absorbance of the control.

### Measurement of confluency and intensities of annexin-V by IncuCyte^®^

Jurkat T cells (1 ×  10^4^/96-well plate) were seeded and treated with the indicated condition for imaging. Before incubation, 1 X annexin-V reagent was added. Differential interference contrast (DIC) microscopic images were obtained by the IncuCyte^®^ imaging system and cellular confluency was calculated by the IncuCyte^®^ software. For annexin-V, microscopic images with green fluorescence for annexin-V were acquired by the IncuCyte^®^ imaging system and the intensities were analyzed by the IncuCyte^®^ software. All intensities were normalized to the intensity of the control and presented in a bar graph.

### Apoptosis assay

Jurkat T cells (1 ×  10^5^/24-well plate) were seeded and treated with the indicated concentrations of lupenone (0 to 40 μM) for 24 h. After incubation, the cells were stained with annexin-V and PI following the manufacturer’s instructions and acquired by flow cytometry to determine the cell populations undergoing apoptosis.

### Sample pre-treatment process in Jurkat T cells

Jurkat T cells (2 ×  10^5^/sample) were pre-treated with 20 μM lupenone or 40 μM lupenone for 1 h and then pretreated with METH for an additional 1 h before CD3/CD28 stimulation.

### Conjugation assay

Jurkat T cells (2 ×  10^5^/sample) stained with CMFDA green fluorescence were pre-treated with 40 μM lupenone for 1 h and exposed to 2 mM METH for 1 h. Raji B cells (2 X 10^5^/sample) stained with CMRA orange fluorescence were pulsed with 1 μg/ml SEE for 30 min. The Jurkat T cells and Raji B cells (1:1) were mixed and incubated for 1 h or 24 h. After incubation, the cells were acquired by flow cytometry to determine the conjugated population (double-positive population) [21].

### Analysis of mRNA levels by conventional and quantitative PCR

After incubation in the indicated conditions, total RNA was isolated using TRIZOL reagent (JBI, Korea). Reverse transcription of the RNA was performed using RT PreMix to obtain cDNA (Enzynomics, Korea). The primers used for each gene were (forward and reverse primers, respectively): human IL2, 5’-CAC GTC TTG CAC TTG TCA C-3’ and 5’-CCT TCT TGG GCA TGT AAA ACT-3’; human CD40L, 5’-GCC AGT TTG AAG GCT TTG TG-3’ and 5’-ACT TAT GAC ATG TGC CGC AA-3’, human GAPDH, 5’-CGG AGT CAA CGG ATT TGG TCG TAT-3’ and 5’-AGC CTT CTC CAT GGT GGT GAA GAC-3’. The conventional PCR conditions were: 30 cycles of denaturation at 94°C for 30 s, annealing at 60°C for 20 s, and extension at 72°C for 40 s, followed by denaturation at 72°C for 7 min. For quantitative PCR analysis, PCR amplification was performed in a DNA Engine Opticon 1 continuous fluorescence detection system (MJ Research, Waltham, MA, USA) using SYBR Premix Ex Taq (Takara, Japan). The total reaction volume was 10 μL, containing 1 μL of cDNA/control and gene-specific primers. Each PCR reaction was performed using the following conditions: 95°C for 30 s and 60°C for 30 s, and then the plate was read (detection of the fluorescent product) for 40 cycles followed by 7 min of extension at 72°C. Melting curve analysis was performed to characterize the dsDNA product by slowly raising the temperature (0.2°C/s) from 60°C to 95°C with fluorescence data collected at 0.2°C intervals. The levels of IL-2 and CD40L mRNA normalized to GAPDH were expressed as fold-changes relative to those of the untreated controls. The fold-change in gene expression was calculated using the following equation: fold change =  2^ − ΔΔCT^, where ΔΔCT =  (CT_target_ - CT_GAPDH_) at time x- (CT_target_ - CT_GAPDH_) at time 0. Here, time x represents any time point and time 0 represents the 1 X expression of the target gene in the untreated cells normalized to GAPDH. Independent experiments were performed at least three times unless otherwise indicated.

### Western blot

Jurkat T cells were incubated in the indicated conditions and lysed by harvesting in RIPA buffer with 1 tablet of phosphatase inhibitor for 30 min on ice. The lysates were centrifuged at 14,000 rpm for 30 min at 4°C and approximately 40 μg of the lysate was separated on 8–12% sodium dodecyl sulfate-polyacrylamide gel electrophoresis (SDS–PAGE) gels. The proteins were transferred to polyvinylidene fluoride (PVDF) membranes (Bio-Rad, Hercules, CA, USA). The membranes were blocked in 5% skim milk for 1 h, rinsed, and incubated with the indicated primary antibodies in 3% skim milk overnight. Excess primary antibodies were removed by washing the membrane three times with Tris-buffered saline-0.1% of Tween (TBS-T) and incubated with 0.1 μg/ml peroxidase-labeled secondary antibodies (against rabbit or mouse) for 1.5 h. After four washes in TBS-T, the bands were visualized with ECL Western blotting detection reagents (Thermo Fisher Scientific, Waltham, MA, USA) using an ImageQuant LAS 4000 (GE Healthcare, Chicago, IL, USA).

### Measurement of surface molecule expression by flow cytometry

The expression of CD69 and CD40L on the T cell surface was analysed by flow cytometry. Jurkat T cells incubated in the indicated conditions were collected and stained with anti-CD69 or anti-CD40L antibodies conjugated to APC. The cells were acquired by flow cytometry and the expressions are presented in a histogram graph. The mean fluorescence intensities are shown in a bar graph.

### IL-2 measurement by ELISA

The amount of IL-2 produced by the cells was determined by ELISA. Cells incubated in the indicated conditions were discarded and the supernatants were collected for ELISA by the DuoSet^®^ ELISA kit following the manufacturer’s instructions.

### Statistics

The mean values ±  SEM were calculated from the data obtained from three independent experiments performed on separate days and presented in the graphs. One-way ANOVA was used to determine significance (P-value). *  indicates significant differences between the indicated groups at P <  0.05.

## Results

### Lupenone is not cytotoxic to Jurkat T cells

**Fig 1:**
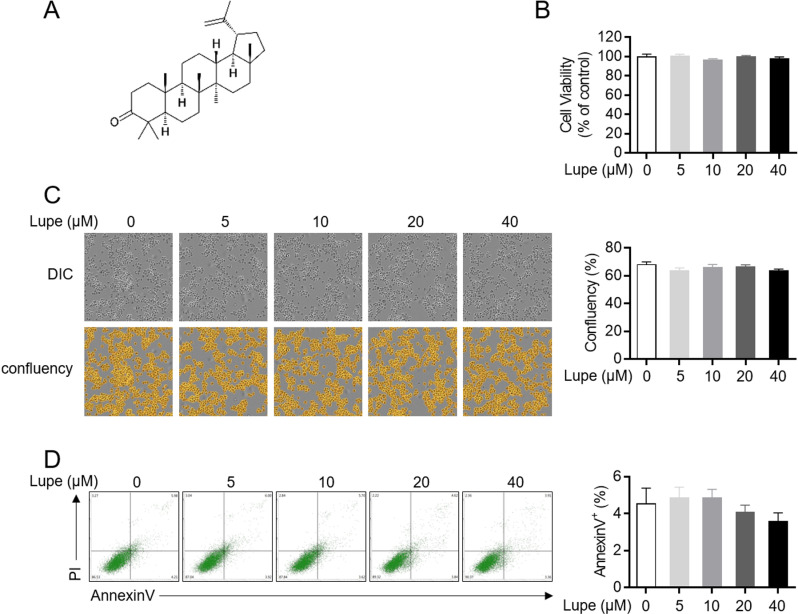
Lupenone is not cytotoxic to Jurkat T cells. (A) The structure of lupenone. (B), (C) Jurkat T cells (5 ×  10^4^/96-well plate) were seeded and treated with the indicated concentrations of lupenone for 24 h. After incubation, microscopic images of the incubated cells were obtained by the IncuCyte^®^ cell imaging system. (B) Cell viability was determined by the MTT assay. (C) Confluent cells were marked in yellow color by the IncuCyte^®^ software and calculated as a percentage (right panel). (D) Jurkat T cells were incubated for Annexin-V/PI apoptosis assay. Annexin-V-positive cells were acquired by flow cytometry and presented as a bar graph in the right panel. The mean value of three experiments ±  SEM is presented.

Even though lupenone (Fig 1A) has previously been shown to be a non-toxic compound, little is known about whether lupenone is toxic to Jurkat T cells. According to a recent report, it showed no cytotoxicity in beta cells (β-cells) and native mouse islet cells at concentrations of 10 µ M and 20 µ M, respectively, and no cytotoxicity in RAW264.7 cells at concentrations of 20 µ M [22, 23]. However, in order to determine the experimental concentration in this study, the cytotoxicity of lupenone was evaluated in Jurkat T, which is not yet known. The MTT assay results supported the findings that cell viability was not affected by treatment with lupenone at any concentration tested (Fig 1B). The DIC images of Jurkat T cells treated with the indicated concentrations of lupenone (0 to 40 μM) for 24 h also revealed that lupenone did not alter the cellular morphology or confluency of the Jurkat T cells (Fig 1C). To confirm whether lupenone induced the apoptotic pathway in Jurkat T cells, an annexin-V/PI assay was performed on Jurkat T cells treated with the indicated concentrations of lupenone for 24 h. The results showed that lupenone treatment did not lead to apoptosis even at 40 μM (Fig 1D). These data indicate that lupenone did not induce cell death, including apoptosis, in Jurkat T cells.

### Lupenone treatment improves T cell activation in METH-exposed cells

**Fig 2:**
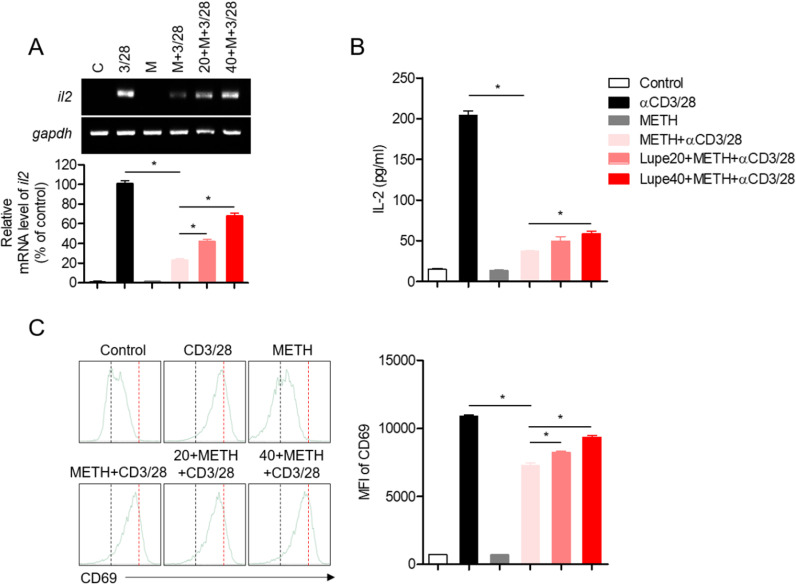
Pre-treatment with lupenone improves T cell activation in METH-exposed cells. (A–C) Jurkat T cells were pre-treated with 0, 20, or 40 μM of lupenone for 1 h and then pre-exposed to 0 or 2 mM METH for 1 h. The cells were then stimulated with anti-CD3/CD28 antibodies for 6 **h** (A), 24 **h** (B), and 16 **h** (C). (A) The IL-2 mRNA level was assessed by quantitative and conventional PCR and normalized to the level of gapdh mRNA. (B) The supernatants were removed for the measurement of IL-2 production by ELISA. (C) The cells were stained with anti-CD69 antibodies to analyze the expression of CD69 on activated T cells. The mean fluorescence intensity was obtained and presented in a bar graph. The mean value of three experiments ±  SEM is presented. * P <  0.05 versus cells pre-exposed to METH and then stimulated with anti-CD3/CD28 antibodies.

METH has been reported to show an immunosuppressive effect on T cell function and activity [9,24]. To elucidate whether lupenone protected T cells from the inhibitory effect of METH exposure on TCR-mediated activation, the mRNA level of IL-2 was measured by quantitative PCR. In addition, IL-2 of T cells was inhibited in Jurkat T cells stimulated with anti-CD3/CD28 antibodies before MeTH stimulation., CD40L, and CD69, and the effects of lupenone on T cell activation markers were confirmed, and it was confirmed that lupenone inhibits T cell activity (S1 Fig). Also, the mRNA analysis results of these factors also showed a significant activation effect compared to the positive control group (S2 Fig). T cells exposed to METH showed reduced IL-2 expression but pre-treatment with lupenone dose-dependently protected IL-2 mRNA levels in the activated condition (Fig 2A). Downregulated IL-2 production was confirmed by ELISA. Pre-treatment with lupenone prevented T cell activation impaired by METH exposure (Fig 2B). To investigate whether pre-treatment with lupenone affected the expression of surface molecules on activated T cells, CD69 expression was determined by flow cytometry. CD69 expression on T cells stimulated with anti-CD3/CD28 antibodies was also protected by lupenone pre-treatment of METH-exposed cells (Fig 2C). These results suggest that T cell activation inhibited by METH exposure was significantly prevented by the pre-treatment of activated T cells with lupenone.

### Pre-treatment with lupenone promotes termination of conjugation with B cells in late phase of immune synapse

**Fig 3:**
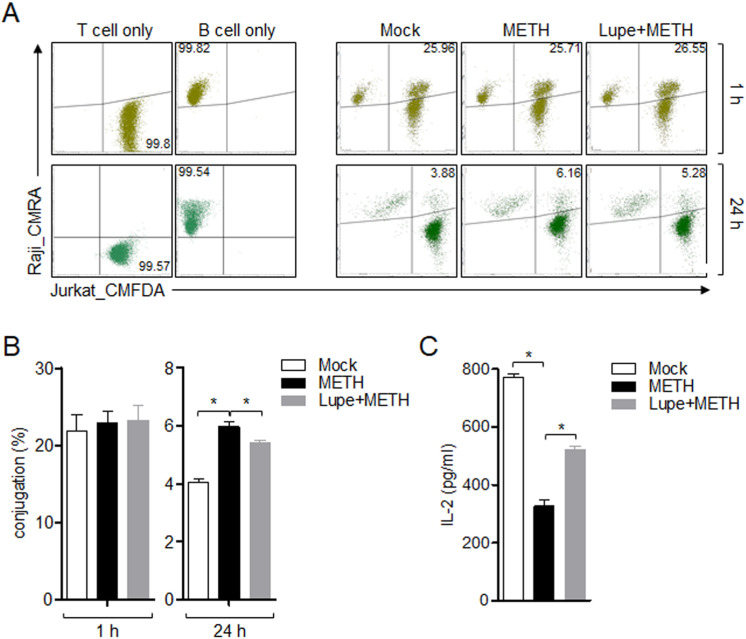
Pre-treatment with lupenone promotes termination of conjugation with B cells in late phase of immune synapse. CMFDA (1 μM)-stained Jurkat T cells were pre-treated with 40 μM of lupenone for 1 h and then pre-exposed to METH for 1 h. (A) CMRA (3 μM)-stained Raji B cells were pulsed with 5 μg/ml of SEE for 30 min and added to the Jurkat T cells for 1 h or 24 h. After incubation, conjugation was analyzed by flow cytometry. The double-positive population indicates conjugated cells and is presented in a bar graph in the right panel. (B) The cell population forming immunological synapse at 1 h and 24 h. (C) The supernatants were collected from 24 h samples to detect IL-2 production by ELISA. The mean value of three experiments ±  SEM is presented. * P <  0.05 between the two groups indicated.

The model of T cell activation using anti-CD3/CD28 antibodies *in vitro* has been considered to mimic T cell activation through the immunological synapse where T cells receive stimulating signals from APCs, including B cells. Besides, conjugation has been investigated to be formed in the early phase of T cell activation but it is maintained to be discontinued in late phase of T cell activation. To elucidate whether METH exposure affected the interaction between T and B cells *in vitro*, a conjugation assay was performed with METH-exposed T cells. Fig 3A shows that early conjugation (within 1 h) between T and B cells was not affected by METH exposure but the termination of immunological formation was prolonged by METH exposure in the later conjugation (24 h). However, pre-treatment with lupenone partially led to termination of the engagement between T and B cells in the late phase of T cell activation (Fig 3B). We assessed the IL-2 production from Jurkat T cells to determine whether the promoting effect of lupenone in termination of conjugation formation influenced T cell activity. As shown in Fig. 3C, the exposure of T cells to METH inhibited the production of IL-2 but pre-treatment with lupenone preserved this function in T cells conjugated with SEE-loaded B cells, consistent with previous data showing that pre-treatment with lupenone enhanced T-B cell interaction (Fig 3A and B). These data suggest that termination of conjugation formation was abnormally regulated in T cells exposed to METH but it could be prevented by pre-treatment with lupenone, leading to T cell activation by B cells.

### Elevated CD40L expression on T cells by pre-treatment with lupenone regulates conjugation of late phase between T and B cells in METH-exposed cells

**Fig 4:**
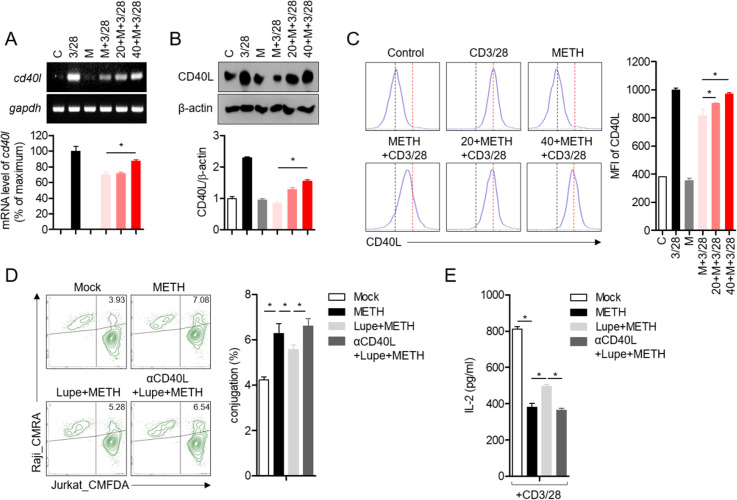
Elevated CD40L expression on T cells by pre-treatment with lupenone enhances conjugation between T and B cells in METH-exposed cells. (A–C) Jurkat T cells were pre-treated with 0, 20, or 40 μM of lupenone for 1 h and then pre-exposed to 0 or 2 mM METH for 1 h. The cells were then stimulated with anti-CD3/CD28 antibodies for 12 **h** (A) and 24 **h** (B, C). (A) The mRNA level of CD40L was assessed by quantitative and conventional PCR. (B) CD40L expression was determined by Western blots. The band intensity was normalized to β-actin and presented in a bar graph. (C) The cells were stained with anti-CD40L anti-bodies to measure the expression of CD40L on activated T cells by flow cytometry. (D, E) The mean fluorescence intensity was obtained and presented in a bar graph. CMFDA (1 μM)-stained Jurkat T cells were pre-treated with 40 μM of lupenone for 1 h and then pre-exposed to METH for 1 h. (D) To neutralize CD40L, antibodies against CD40L were added. CMRA (3 μM)-stained Raji B cells were pulsed with 5 μg/ml of SEE for 30 min and added to Jurkat T cells for 24 h. The double-positive population indicates conjugated cells and is presented in a bar graph in the right panel. (E) The supernatants were removed and IL-2 production was assessed by ELISA. The mean value of three experiments ±  SEM is presented. * P <  0.05 between the two groups indicated.

To determine the underlying mechanism how immunological synapse are controlled in the late phase of T cell activation, we investigated whether CD40L, one of the critical surface molecules in immunological synapses, was involved in this process. The mRNA level of CD40L in activated T cells pre-exposed to METH was significantly reduced but a dose-dependent preserved by pre-treatment with lupenone was seen (Fig 4A). The protein level of CD40L was also confirmed by Western blots and flow cytometry. CD40L was prevented by pre-treatment with lupenone in METH-exposed cells (Fig 4B and C). To explore whether the protected expression of CD40L by pre-treatment with lupenone affected the conjugation of T cells with B cells and the activity of the T cells, we assessed the engagement between T and B cells and IL-2 production using anti-CD40L neutralizing anti-bodies. T cells conjugated with B cells was suppressed in the presence of lupenone in METH-exposed cells but blockade of CD40L by anti-CD40L neutralizing antibodies elevated conjugation (Fig 4D). Furthermore, IL-2 production was partially enhanced in T cells conjugated with B cells pre-treated with lupenone in METH exposure condition but anti-CD40L neutralizing antibodies blocked the protective effect of lupenone (Fig 4E). These results suggest that the expression of CD40L was preserved by pre-treatment with lupenone and its role was critical for the regulation of interaction of T-B cells and T cell activity under METH exposure conditions.

### Lupenone treatment protects T cells from apoptosis induced by METH exposure

**Fig 5:**
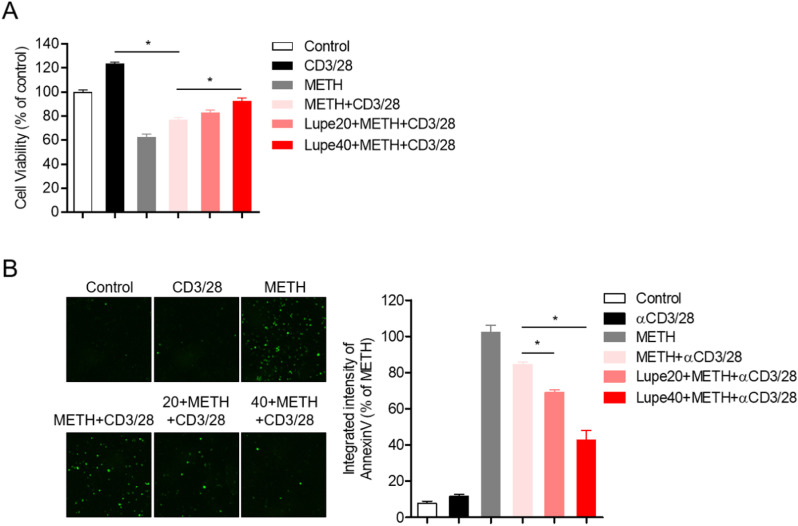
Pre-treatment with lupenone protects T cells from apoptosis induced by METH exposure. (A, B) The staining reagent for annexin-V (green fluorescence) was added to the Jurkat T cells. Cells were pre-treated with 0, 20, or 40 μM of lupenone for 1 h and then pre-exposed to 0 or 2 mM of METH for 1 h. Then, the cells were stimulated with anti-CD3/CD28 antibodies for 24 h. (A) Cell viability was determined by the MTT assay. (B) Microscopic images with fluorescence indicating annexin-V (green) were obtained by the IncuCyte^®^ cell imaging system. The fluorescence intensities were integrated by the IncuCyte^®^ software and are presented in a bar graph (right panel). The mean value of three experiments ±  SEM is presented. * P <  0.05 versus cells pre-exposed to METH and stimulated with anti-CD3/CD28 antibodies.

METH exposure is known to cause cytotoxicity and initiate apoptosis through the PI3K/Akt/mTOR survival pathway [3,4]. Since several reports have revealed that apoptosis had a regulatory effect on T cell activity, we explored whether the cytotoxicity induced by METH was involved in T cell activation and the protective role of pre-treatment with lupenone in this condition. Reduced cell viability was seen in TCR-stimulated T cells exposed to METH but the decrease in viability was protected by pre-treatment with lupenone in a dose-dependent manner (Fig 5A). To confirm whether pre-treatment with lupenone suppressed apoptotic pathways induced by METH in activated T cells, the expression of annexin-V was detected by the IncuCyte^®^ cell imaging system. Fig 5B shows the elevated expression of annexin-V in T cells exposed to METH and stimulated with anti-CD3/CD28 antibodies but partially reduced expression in T cells pre-treated with lupenone. These results demonstrated that pre-treatment with lupenone suppresses cytotoxicity of METH on activated T cells.

### Lupenone increases the expression of anti-apoptotic proteins in activated T cells exposed to METH

**Fig 6:**
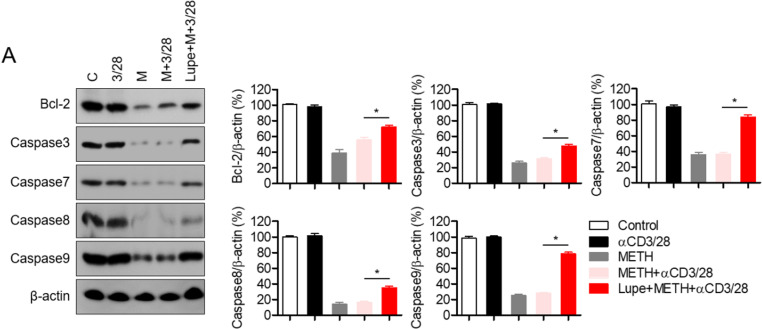
Lupenone prevents the expression of anti-apoptotic proteins in activated T cells exposed to METH. Jurkat T cells were pre-treated with 40 μM of lupenone for 1 h and then pre-exposed to 2 mM of METH for 1 h. (A) The cells were then stimulated with anti-CD3/CD28 antibodies for 24 h and harvested for Western blot analysis. The expressions of the indicated proteins were detected, normalized to β-actin, and presented in a bar graph. The mean value of three experiments ±  SEM is presented. * P <  0.05 versus cells pre-exposed to METH and stimulated with anti-CD3/CD28 antibodies.

Apoptosis is tightly regulated by the balance of pro-apoptotic and anti-apoptotic proteins [23]. Since molecular changes in anti-apoptotic proteins are induced by METH exposure [20], the expression of anti-apoptotic proteins was determined by Western blot analysis. Suppressed expression of Bcl-2, caspase 8, and caspase 9 was detected in METH-exposed T cells but the decreases were significantly protected by pre-treatment with lupenone. Also, it was confirmed that the expression of caspase 3 and caspase 7 was also protected by lupenone treatment in T cells exposed to METH (Fig 6A). Therefore, these data suggest that pre-treatment with lupenone confered protection from cytotoxicity and apoptosis induced by METH exposure by restoring the expression of anti-apoptotic proteins.

### Protective effects of lupenone on activated T cells exposed to METH are associated with the recovery of MAPK and PI3K/Akt/mTOR signaling pathways

**Fig 7:**
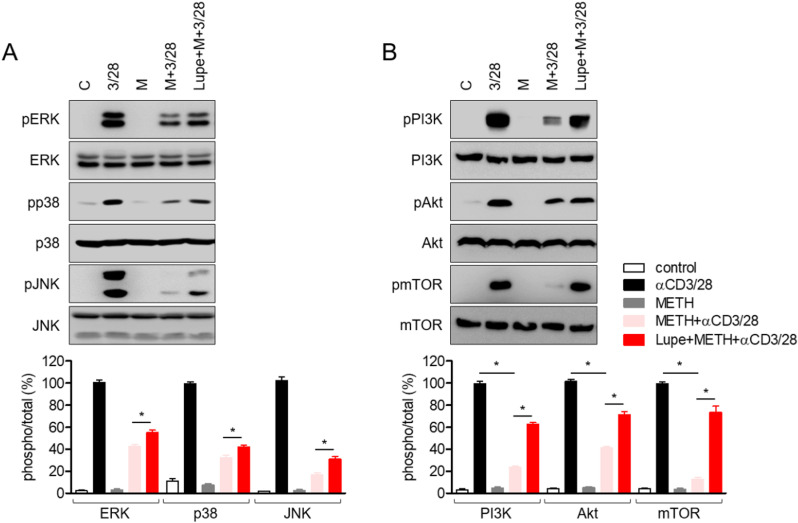
Protective effects of lupenone on activated T cells exposed to METH are associated with the protection of MAPK and PI3K/Akt/mTOR signaling pathways. (A, B) Starved Jurkat T cells in plain media were pre-treated with 40 μM of lupenone for 1 h and then pre-exposed to 2 mM of METH for 1 h. The cells were then stimulated with anti-CD3/CD28 antibodies for 30 min and harvested for Western blot analysis. The phosphorylation levels of the indicated proteins were detected and normalized to the amount of the total form of the indicated proteins and presented in a bar graph. The mean value of three experiments ±  SEM is presented. * P <  0.05 versus cells pre-exposed to METH and stimulated with anti-CD3/CD28 antibodies.

Since the MAPK signaling pathway is mainly involved in T cell activation stimulated by anti-CD3/CD28 antibodies, we investigated whether pre-treatment with lupenone accelerated MAPK signaling under METH exposure conditions. The phosphorylation levels of ERK, p38, and JNK were preserved in activated T cells pre-treated with lupenone in METH-exposed cells (Fig 7A). Additionally, treatment with lufenone showed an effect on the phosphorylation processes of PI3K, Akt, and mTOR during T cell activation stimulated by anti-CD3/CD28 antibodies (Fig 7B).

## Discussion

The present study revealed that lupenone isolated from *S. commixta* had a protective effect on the T cell activation and viability of stimulated T cells under METH exposure conditions. Lupenone, non-toxic to Jurkat T cells, protected IL-2 production and CD69 expression in activated T cells, which were suppressed by exposure to METH. Pre-treatment with lupenone partially prevented the inhibitory effect of METH on the termination of conjugation between T and B cells by preserving CD40L expression on activated T cells. However, several cytotoxicity assays showed that pre-treatment with lupenone increased the viability of METH-exposed T cells in a dose-dependent manner. Western blot analysis showing that the reduced expression of anti-apoptotic proteins and PI3/Akt/mTOR signaling pathways by METH exposure were prevented by pre-treatment with lupenone supported these processes *in vitro*.

Because T cells play a pivotal role in immunity as well as immunopathology, the activation of T cells is strictly controlled by several molecules involved in apoptosis and the cell death pathway [25]. The contribution of apoptosis to the regulation of T cell function has been investigated. Dysregulation of the cell death pathway leads to several immunological diseases, including autoimmunity or immunodeficiency [26]. It has been reported that patients who abuse METH have lowered resistance to viral and microbial infections [9,27] and T cells treated with METH showed decreased proliferative capacity compared to untreated T cells [8]. In the present study, we elucidated the protective effect of lupenone against the METH-induced cytotoxicity of activated T cells, which led to the protection of IL-2 production and CD69 expression. These results suggest that the prevented T cell activation by pre-treatment with lupenone resulted from the enhanced expression of CD40L as well as the reduced cytotoxicity of METH-exposed T cells. Further studies should include *in vivo* experiments showing whether the oral administration of lupenone attenuates immunodeficiency in the METH addiction mouse model.

The role of CD40L in cellular responses has been investigated for a long time. Specifically, how the signaling pathway generated by CD40L affects apoptotic signaling has been well-explored. The therapeutic potential of CD40L as a target of anticancer drugs was demonstrated by the promotion of apoptosis by CD40L-CD40 interaction in carcinoma cells [28]. However, conflicting reports have shown that CD40L-CD40 interaction effectively suppressed apoptotic signaling induced by TNFα in osteoblasts [29]. These conflicting findings may suggest the complexity of CD40L-CD40 signaling and how this interaction plays different roles in cell responses in situation-dependent fashions. T cells are considered to have the most well-defined roles of CD40L-CD40 interaction. It was investigated that CD40L on T cells provides survival signals in the immune synapse with B cells and protects Fas-mediated apoptosis by induction of anti-apoptotic proteins expression [30]. In the present study, our results revealed that the pre-treatment of activated T cells with lupenone promoted CD40L expression and termination of conjugation with B cells. The expression of anti-apoptotic proteins, including Bcl-2 and the caspase family, was protected by upregulation of CD40L expression by pre-treatment with lupenone in METH-exposed cells. These results suggest that lupenone treatment may promote CD40L expression to protect T cells from apoptotic signals by simultaneously increasing the expression of anti-apoptotic proteins in METH-exposed cells.

For the initiation and generation of the immune response, antigen-dependent or an-antigen-independent interactions between ligands and receptors are involved in T cell priming. T cells are primed by dendritic cells to induce CD40L expression on their surface and assist B lymphocyte differentiation into plasma cells, generating antibodies through CD40L-CD40 conjugation [31]. Specifically in the late phase of T cell activation, stimulated T cells are separated from the immunological synapse to proliferate and differentiate into memory T cells [32]. Knockout mice studies have reported the importance of CD40L in the antiviral immune response [33] or in generating humoral immunity from plasma cells [34]. CD40L deficiency and mutations in the CD40L gene have been shown to cause severe immunodeficiency called X-linked hyper-IgM syndrome and patients with CD40L-deficiency are susceptible to severe infections [35, 36].

In the current study, we showed that CD40L expression was significantly reduced by METH exposure but partially protected in the presence of lupenone. The results showing IL-2 production indicated that this prevention by lupenone led to enhanced T cell activation in METH-exposed cells. Elucidation of the underlying molecular mechanism between METH exposure and the expression of CD40L on activated T cells would provide us with a potential strategy for developing therapeutics and implicate lupenone as an inducer of CD40L expression.

## Supporting information

S1 Fig Effect of lufenone on anti-CD3 CD28 antibody stimulation.
(DOCX)

S2 Fig Comparison of the effects of lufenone and positive control on anti-CD3 CD28 antibody stimulation.
(DOCX)

S1 File Original image.
(PDF)

## References

[1] Colfax G, Shoptaw S. The methamphetamine epidemic: implications for HIV prevention and treatment. Curr HIV/AIDS Rep. 2005;2(4):194–9. doi: 10.1007/s11904-005-0016-4 16343378

[2] Martinez LR, Mihu MR, Gácser A, Santambrogio L, Nosanchuk JD. Methamphetamine enhances histoplasmosis by immunosuppression of the host. J Infect Dis. 2009;200(1):131–41. doi: 10.1086/599328 19473099 PMC11530355

[3] Granado N, Ares-Santos S, Moratalla R. Methamphetamine and Parkinson’s disease. Parkinsons Dis. 2013;2013308052. doi: 10.1155/2013/308052 23476887 PMC3582059

[4] Yu S, Zhu L, Shen Q, Bai X, Di X. Recent advances in methamphetamine neurotoxicity mechanisms and its molecular pathophysiology. Behav Neurol. 2015;2015103969. doi: 10.1155/2015/103969 25861156 PMC4377385

[5] Mahajan SD, Hu Z, Reynolds JL, Aalinkeel R, Schwartz SA, Nair MPN. Methamphetamine modulates gene expression patterns in monocyte derived mature dendritic cells: implications for HIV-1 pathogenesis. Mol Diagn Ther. 2006;10(4):257–69. doi: 10.1007/BF03256465 16884330

[6] Nair MPN, Saiyed ZM, Nair N, Gandhi NH, Rodriguez JW, Boukli N, et al. Methamphetamine enhances HIV-1 infectivity in monocyte derived dendritic cells. J Neuroimmune Pharmacol. 2009;4(1):129–39. doi: 10.1007/s11481-008-9128-0 18958626 PMC3764920

[7] Tallóczy Z, Martinez J, Joset D, Ray Y, Gácser A, Toussi S, et al. Methamphetamine inhibits antigen processing, presentation, and phagocytosis. PLoS Pathog. 2008;4(2):e28. doi: 10.1371/journal.ppat.0040028 18282092 PMC2242831

[8] Potula R, Haldar B, Cenna JM, Sriram U, Fan S. Methamphetamine alters T cell cycle entry and progression: role in immune dysfunction. Cell Death Discov. 2018;4 44. doi: 10.1038/s41420-018-0045-6 29581895 PMC5859078

[9] Yu Q, Zhang D, Walston M, Zhang J, Liu Y, Watson RR. Chronic methamphetamine exposure alters immune function in normal and retrovirus-infected mice. Int Immunopharmacol. 2002;2(7):951–62. doi: 10.1016/s1567-5769(02)00047-4 12188036

[10] Dustin ML. T-cell activation through immunological synapses and kinapses. Immunol Rev. 2008;221 77–89. doi: 10.1111/j.1600-065X.2008.00589.x 18275476

[11] Howland KC, Ausubel LJ, London CA, Abbas AK. The roles of CD28 and CD40 ligand in T cell activation and tolerance. J Immunol. 2000;164(9):4465–70. doi: 10.4049/jimmunol.164.9.4465 10779746

[12] Aversa G, Punnonen J, Carballido JM, Cocks BG, de Vries JE. CD40 ligand-CD40 interaction in Ig isotype switching in mature and immature human B cells. Semin Immunol. 1994;6(5):295–301. doi: 10.1006/smim.1994.1038 7532459

[13] Gutierrez-Lugo M-T, Deschamps JD, Holman TR, Suarez E, Timmermann BN. Lipoxygenase inhibition by anadanthoflavone, a new flavonoid from the aerial parts of Anadenanthera colubrina. Planta Med. 2004;70(3):263–5. doi: 10.1055/s-2004-818920 15114507

[14] Na M, Kim BY, Osada H, Ahn JS. Inhibition of protein tyrosine phosphatase 1B by lupeol and lupenone isolated from Sorbus commixta. J Enzyme Inhib Med Chem. 2009;24(4):1056–9. doi: 10.1080/14756360802693312 19548777

[15] Kim H, Song M-J. Traditional plant-based therapies for respiratory diseases found in North Jeolla Province, Korea. J Altern Complement Med. 2012;18(3):287–93. doi: 10.1089/acm.2010.0848 22394156

[16] Kim H, Song M-J. Ethnomedicinal practices for treating liver disorders of local communities in the southern regions of Korea. Evid Based Complement Alternat Med. 2013;2013869176. doi: 10.1155/2013/869176 24089622 PMC3782137

[17] Xu F, Huang X, Wu H, Wang X. Beneﬁcial health effects of lupenone triterpene: A review. Biomed Pharmacother. 2018;103 198–203. doi: 10.1016/j.biopha.2018.04.019 29653365

[18] Xu F, Wu H, Wang X, Yang Y, Wang Y, Qian H, et al. RP-HPLC characterization of lupenone and β-sitosterol in rhizoma musae and evaluation of the anti-diabetic activity of lupenone in diabetic Sprague-Dawley rats. Molecules. 2014;19(9):14114–27. doi: 10.3390/molecules190914114 25207716 PMC6270966

[19] Xu F, Yang L, Huang X, Liang Y, Wang X, Wu H. Lupenone is a good anti-inflammatory compound based on the network pharmacology. Mol Divers. 2020;24(1):21–30. doi: 10.1007/s11030-019-09928-5 30796639

[20] Lee H-S, Kim E-N, Jeong G-S. Lupenone Protects Neuroblastoma SH-SY5y Cells Against Methamphetamine-Induced Apoptotic Cell Death via PI3K/Akt/mTOR Signaling Pathway. Int J Mol Sci. 2020;21(5):1617. doi: 10.3390/ijms21051617 32120831 PMC7084488

[21] Na BR, Jun CD. In vitro Assessment of Immunological Synapse Formation by Flow Cytometry. Bio-protocol. 2016;6(6):e1758. doi: 10.21769/BioProtoc.1758 1758

[22] Song SE, Shin SK, Kim YW, Do YR, Lim AK, Bae JH, et al. Lupenone attenuates thapsigargin-induced endoplasmic reticulum stress and apoptosis in pancreatic beta cells possibly through inhibition of protein tyrosine kinase 2 activity. Life Sciences. 2023;332. doi: 10.1016/j.lfs.2023.122107 37739164

[23] Jin SE, Son YK, Min B-S, Jung HA, Choi JS. Anti-inflammatory and antioxidant activities of constituents isolated from Pueraria lobata roots. Arch Pharm Res. 2012;35(5):823–37. doi: 10.1007/s12272-012-0508-x 22644850

[24] Mata MM, Napier TC, Graves SM, Mahmood F, Raeisi S, Baum LL. Methamphetamine decreases CD4 T cell frequency and alters pro-inflammatory cytokine production in a model of drug abuse. Eur J Pharmacol. 2015;752 26–33. doi: 10.1016/j.ejphar.2015.02.002 25678251 PMC4396630

[25] Fulda S. Shifting the balance of mitochondrial apoptosis: therapeutic perspectives. Front Oncol. 2012;2 121. doi: 10.3389/fonc.2012.00121 . 23061040 PMC3465793

[26] Zhan Y, Carrington EM, Zhang Y, Heinzel S, Lew AM. Life and Death of Activated T Cells: How Are They Different from Naïve T Cells?. Front Immunol. 2017;8 1809. doi: 10.3389/fimmu.2017.01809 29326701 PMC5733345

[27] Zhang N, Hartig H, Dzhagalov I, Draper D, He YW. The role of apoptosis in the development and function of T lymphocytes. Cell Res. 2005;15(10):749–69. doi: 10.1038/sj.cr.7290345 16246265

[28] Harms R, Morsey B, Boyer CW, Fox HS, Sarvetnick N. Methamphetamine administration targets multiple immune subsets and induces phenotypic alterations suggestive of immunosuppression. PLoS One. 2012;7(12):e49897. doi: 10.1371/journal.pone.0049897 23227154 PMC3515581

[29] Eliopoulos AG, Davies C, Knox PG, Gallagher NJ, Afford SC, Adams DH, et al. CD40 induces apoptosis in carcinoma cells through activation of cytotoxic ligands of the tumor necrosis factor superfamily. Mol Cell Biol. 2000;20(15):5503–15. doi: 10.1128/MCB.20.15.5503-5515.2000 10891490 PMC86001

[30] Ahuja SS, Zhao S, Bellido T, Plotkin LI, Jimenez F, Bonewald LF. CD40 ligand blocks apoptosis induced by tumor necrosis factor alpha, glucocorticoids, and etoposide in osteoblasts and the osteocyte-like cell line murine long bone osteocyte-Y4. Endocrinology. 2003;144(5):1761–9. doi: 10.1210/en.2002-221136 12697681

[31] Elgueta R, Benson MJ, de Vries VC, Wasiuk A, Guo Y, Noelle RJ. Molecular mechanism and function of CD40/CD40L engagement in the immune system. Immunol Rev. 2009;229(1):152–72. doi: 10.1111/j.1600-065X.2009.00782.x 19426221 PMC3826168

[32] Marasco E, Farroni C, Cascioli S, Marcellini V, Scarsella M, Giorda E, et al. B-cell activation with CD40L or CpG measures the function of B-cell subsets and identifies specific defects in immunodeficient patients. Eur J Immunol. 2017;47(1):131–43. doi: 10.1002/eji.201646574 27800605

[33] Ara A, Ahmed KA, Xiang J. Multiple effects of CD40-CD40L axis in immunity against infection and cancer. Immunotargets Ther. 2018;7 55–61. doi: 10.2147/ITT.S163614 29988701 PMC6029590

[34] Borrow P, Tishon A, Lee S, Xu J, Grewal IS, Oldstone MB, et al. CD40L-deficient mice show deficits in antiviral immunity and have an impaired memory CD8+ CTL response. J Exp Med. 1996;183(5):2129–42. doi: 10.1084/jem.183.5.2129 8642323 PMC2192549

[35] Whitmire JK, Slifka MK, Grewal IS, Flavell RA, Ahmed R. CD40 ligand-deficient mice generate a normal primary cytotoxic T-lymphocyte response but a defective humoral response to a viral infection. J Virol. 1996;70(12):8375–81. doi: 10.1128/JVI.70.12.8375-8381.1996 8970958 PMC190926

[36] França TT, Barreiros LA, Al-Ramadi BK, Ochs HD, Cabral-Marques O, Condino-Neto A. CD40 ligand deficiency: treatment strategies and novel therapeutic perspectives. Expert Rev Clin Immunol. 2019;15(5):529–40. doi: 10.1080/1744666X.2019.1573674 . 30681380

