# Supplementary material for: Lupenone preserves T cell activity by recovery of CD40L expression and protection from cytotoxicity due to methamphetamine exposure
Source: PLoS One. 2025 Mar 20;20(3):e0314054. doi: 10.1371/journal.pone.0314054 (PMC11925290; doi:10.1371/journal.pone.0314054)
Supplement: S1 Fig — (DOCX) [file pone.0314054.s001.docx]

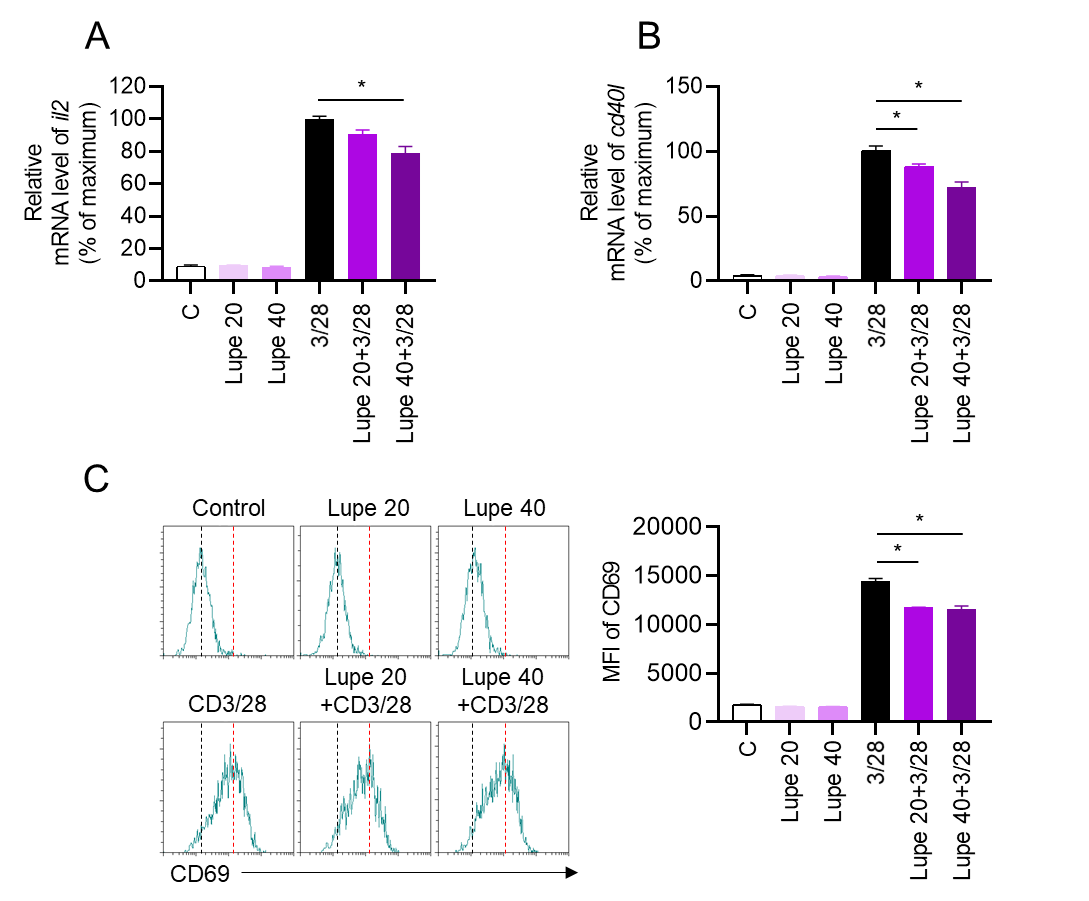


**S1 Fig. Effect of lufenone on anti-CD3CD28 antibody stimulation**. (A-C) Jurkat T cells were pre-treated with 0, 20, or 40 μM of lupenone for 1 h and then stimulated with anti-CD3/CD28 antibodies for 6 h (A), 12 h (B), and 16 h (C). The IL-2 (A) and CD40L (B) mRNA level was assessed by quantitative PCR and normalized to the level of GAPDH mRNA. The cells were stained with anti-CD69 antibodies to analyze the expression of CD69 on activated T cells (C). The mean fluorescence intensity was obtained and presented in a bar graph. The mean value of three experiments ± SEM is presented. **P* < 0.05 versus cells stimulated with anti-CD3/CD28 antibodies.
