# Supplementary material for: Lupenone preserves T cell activity by recovery of CD40L expression and protection from cytotoxicity due to methamphetamine exposure
Source: PLoS One. 2025 Mar 20;20(3):e0314054. doi: 10.1371/journal.pone.0314054 (PMC11925290; doi:10.1371/journal.pone.0314054)
Supplement: S2 Fig — (DOCX) [file pone.0314054.s002.docx]

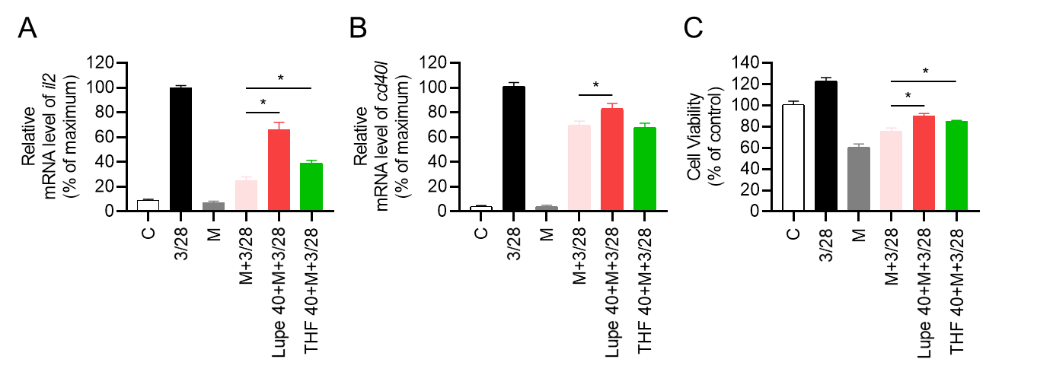


**S2 Fig. Comparison of the effects of lufenone and positive control on anti-CD3CD28 antibody stimulation.** (A-C) Jurkat T cells were pre-treated with 40 μM of lupenone or THF for 1 h and then pre-exposed to 2 mM METH for 1 h. The cells were then stimulated with anti-CD3/CD28 antibodies for 6 h (A), 12 h (B), and 24 h (C). The IL-2 (A) or CD40L (B) mRNA level was assessed by quantitative PCR and normalized to the level of GAPDH mRNA. Cell viability was determined by the MTT assay (C). The mean value of three experiments ± SEM is presented. **P* < 0.05 versus cells pre-exposed to METH and then stimulated with anti-CD3/CD28 antibodies.
