# Supplementary figures and images for: Lupenone preserves T cell activity by recovery of CD40L expression and protection from cytotoxicity due to methamphetamine exposure
Source: PLoS One. 2025 Mar 20;20(3):e0314054. doi: 10.1371/journal.pone.0314054 (PMC11925290; doi:10.1371/journal.pone.0314054)

## S1 File: Original image

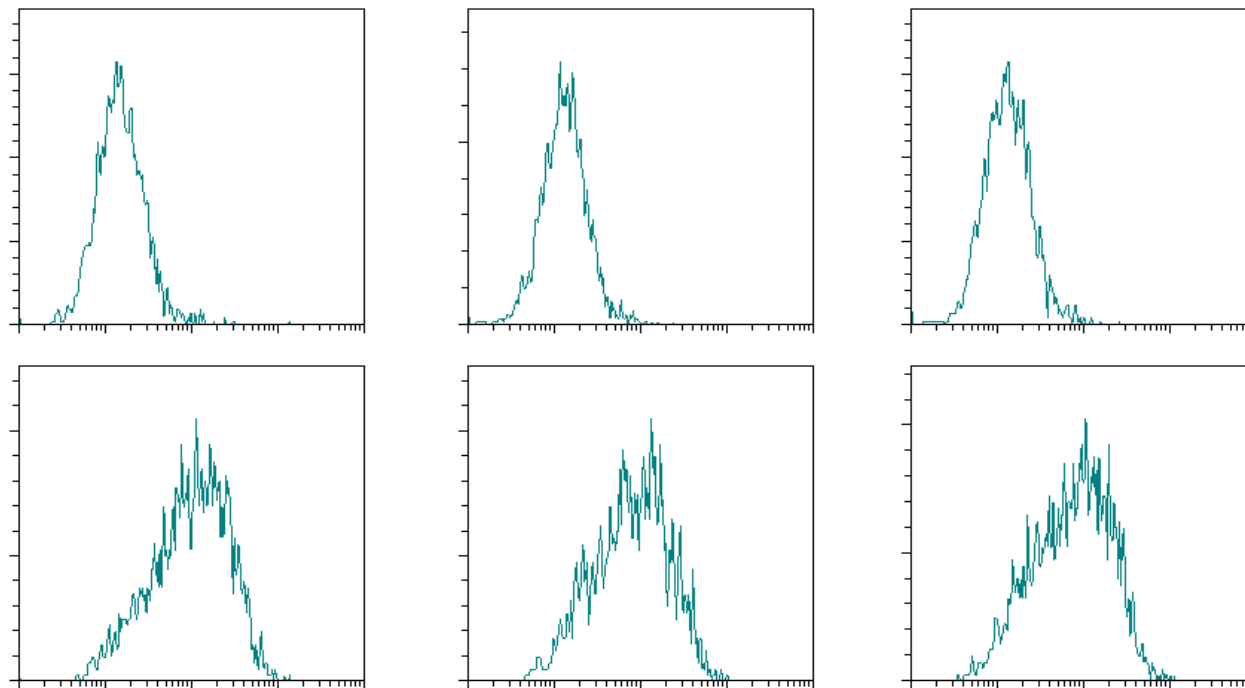

Supplement: S1 File — (PDF) [file pone.0314054.s003.pdf]
